# Death from Inoculation of Virous from a Cancer

**Published:** 1860-10

**Authors:** 


*Death from Inoculation of Virous from a Cancer.*—D. Lemarchand died in consequence of a puncture with a suture needle, which had laid some time in a wound made by the removal of a cancerous tumor. B.

